# Human and canine visceral leishmaniasis in an emerging focus in Araçuaí, Minas Gerais: spatial distribution and socio-environmental factors

**DOI:** 10.1590/0074-02760160133

**Published:** 2016-07-04

**Authors:** Renata Luiz Ursine, João Victor Leite Dias, Harriman Aley Morais, Herton Helder Rocha Pires

**Affiliations:** Universidade Federal dos Vales do Jequitinhonha e Mucuri, Grupo de Extensão e Pesquisa em Saúde Coletiva, Diamantina, MG, Brasil

**Keywords:** kala-azar, environmental health, urbanisation, spatial analysis

## Abstract

This study aimed to analyse the spatial distribution of human (2007-2013) and canine (2013) visceral leishmaniasis (VL) in the city of Araçuaí, Minas Gerais, Brazil, and identify the socio-environmental factors related to their occurrence. The spatial distribution of human and canine cases was analysed by kernel density estimation (KDE) and the K function. The KDE values were analysed for correlation between human and canine LV and for normalised difference vegetation index (NDVI). Socio-environmental aspects of household structures and surroundings were evaluated. The spatial distribution of human and canine VL cases exhibited a significant aggregated pattern in distances greater than 350 and 75 m, respectively. The higher occurrence of human and canine infection occurred in the central area of the city. A positive correlation between the densities of human and canine cases was observed, as well as a negative correlation between NDVI and densities of human and canine cases. Socio-environmental analysis revealed that the large amount of animals, organic material from trees and deficiencies in environmental sanitation are possibly contributing to the continuation of the transmission cycle of *Leishmania infantum* in Araçuaí. These results can contribute to the planning by competent agencies to reduce the incidence of infection in the city.

Visceral leishmaniasis (VL) has a large geographic distribution, occurring in Asia, Europe, the Middle East, Africa and the Americas, affecting 88 countries, the majority of which are classified as developing. It is estimated that approximately 200,000 to 400,000 new cases occur every year, with 20,000 to 40,000 of these being fatal (Alvar et al. 2012).

In Brazil, VL has caused severe public health problems, due to its difficulty to control, high lethality and wide distribution, as it occurs within the five regions of the country (do Prado et al. 2011). In recent years, this endemic has shown a clear expansion and urbanisation related to environmental, social and demographic factors, in addition to the adaptation of *Lutzomyia longipalpis* to the urban environment and the presence of domestic reservoirs (de Almeida et al. 2011, Cardim et al. 2013, Marcondes & Rossi 2013, Ortiz & Anversa 2015, Mendes et al. 2016).

The Brazilian VL control program recommends the diagnosis and treatment of infected individuals, identification and control of domestic reservoirs, vector control, environmental management and health education services (MS/SVS 2006). The success of these program activities, however, is variable depending on the condition of each city and the way in which they are executed (Carneiro et al. 2013, Morais et al. 2015).

Considering this, the use of geotechnology, such as spatial statistics and remote sensing, has contributed to the investigation of socio-environmental aspects related to VL, as it generates data that can possibly identify priority locales for development of vigilance and control programs (Cardim et al. 2013, Barbosa et al. 2014, Borges et al. 2014, de Almeida et al. 2014, Teixeira-Neto et al. 2014, Vieira et al. 2014).

The city of Araçuaí, located in the valley of Jequiti- nhonha, Minas Gerais, Brazil, is endemic for VL (Santos et al. 2013), but many aspects related to this phenomenon are still poorly described. Given that, this study aimed to analyse the spatial distribution of human and canine VL and the socio-environmental factors related to the areas of human infection occurrence within the city.

## MATERIALS AND METHODS

*Study area* - The city of Araçuaí (lat: - 16.85; long: - 42.06) is located in the middle Jequitinhonha Valley, in the state of Minas Gerais, and lies 289 m above sea level (INMET/MAPA 2015). The municipality occupies an area of 2,236.279 km^2^, with an estimated population of 37,220 inhabitants in 2015 (MP/IBGE 2015).

The climate is semi-arid, with 823.1 mm average annual precipitation, with the majority of rainfall concentrated during the period from October-March. The average annual temperature is around 24.2ºC (14.9ºC-33.4ºC), with February the hottest month, and July the coldest (INMET/MAPA 2015).

Principal economic activities in Araçuaí are related to subsistence agriculture, handicraft manufacture and informal mining (MP/IBGE 2015).

*Human and canine VL data* - Notification data related to cases of human infection by *Leishmania infantum (= Leishmania chagasi)* in Araçuaí were collected from records of the Information System on Notifiable Diseases in May, 2014, regarding the period from 2007-2013. Among the required registration fields, data were collected regarding sex, age and residence zone. Among the essential but not required registration fields, data regarding the evolution of the cases were collected.

The annual incidence of VL in the city was calculated considering the population at risk estimated from the population data from the 1991, 2000 and 2010 demographic censuses, and population counts of 1996 and 2007, using an equation of geometric growth model. We adopted this model because it presented the best fit to the population data, with a higher determination coefficient (R^2^ = 0.9025) than linear, exponential and logarithmic models.

To evaluate the relationship between the presence of human infection by *L. infantum* and occurrence areas of canine infection, data from serological canine exams from surveys conducted in 2013 were acquired from records available in the Araçuaí Secretary of Health. According to the health service routine, these surveys were carried out by sampling, using a rapid immunochromatographic test (Dual-Path Platform technology - DPP®) and the Enzyme-linked Immunosorbent Assay (ELISA). The following information was collected from the records: locality, date of exam and its result.

*Spatial analyses* - The households of people who had VL during the period of the study were georeferenced using handheld GPS equipment (Garmin Oregon^TM^). Canine infections for 2013 were referenced based on centroids of blocks in which they occurred, in agreement with sketches from the city’s health service, designed from composition of images in Google Earth®. The reference projection used was the UTM, zone 23 S, *datum* WGS 84.

The spatial distribution analysis of human and canine VL was performed using the quartic function of kernel density estimation (KDE) (Gatrell et al. 1996), considering a bandwidth of 400 m, and generating a matrix with a grid cell size of 30 m. The KDE is a non-parametric function that estimates the values of probable occurrence based on the density of events nearby. For human cases of VL we used only quartic function that generated an image with values of estimated density by each pixel. For canine cases we followed the same procedure, but added a multiplier attribute (proportion of positive dogs in each block) to the KDE in order to weight the function.

The spatial aggregation of infection was analysed by the K function (Ripley 1981), whose statistical significance was evaluated using 999 permutations in a Complete Spatial Randomness model, considering a significance level of 0.05. The spatial statistical analyses were only conducted for the urban area of Araçuaí, and were performed using SPRING 5.2.7 (Câmara et al. 1996).

The normalised difference vegetation index (NDVI) was adopted to evaluate correlation between the quantity of vegetation and the occurrence of VL in Araçuaí. NDVI was calculated by means of bands 3 (red) and 4 (near infrared) of the Thematic Mapper sensor on the LANDSAT 5 satellite, with 30 m of spatial resolution. The images used for the calculation corresponded to orbit 217, point 072, collected on July 1, 2007, August 4, 2008, August 7, 2009 and August 26, 2010. These images were selected for their low cloud cover of the study area. All of the images were georeferenced based on an orthorectified image and were corrected for atmospheric effects by means of dark pixel subtraction (Chavez Jr 1988). NDVI was calculated for each of the images and the average index over the four year period was used for analysis.

The satellite images were obtained from the Brazilian Institute for Space Research (http://www.inpe.br) and the United States Geological Survey (earthexplorer.usgs.gov/). Image processing was performed with SPRING 5.2.7.

The matrices of NDVI values were exported in a text document and the grid cells were superimposed and paired with KDE values for human and canine VL to evaluate the correlation between variables. The Spearman correlation coefficient (ρ) was used in GraphPad Prism 5.0® (GraphPad Software, San Diego, CA).

*Description of housing and surroundings* - Socio-environmental aspects of housing structures and their surroundings were evaluated using a structured form based on a detailed questionnaire designed by Fernandes (2012) for the study of environmental aspects associated with prevalence of canine infection by *L. infantum* in Teresina, Piauí. All the houses with reported cases of human VL were visited by a member of the research team (between February 2014 and June 2014) that fulfilled the form based on personal observation and the responses provided by householder.

*Statistical analyses* - The variables sex, age group by sex and residence zone were analysed using a corrected *X* ^2^ test (Yates) with expected equal rates, considering a significance level of 0.05 in BioEstat 5.0 (Ayres et al. 2007).

This study was approved by the Committee on Ethics in Research of the Federal University of Vales do Jequiti- nhonha and Mucuri, under number 522.035, respecting Resolution 466/12 of the National Board of Health. All participants in the research signed the Terms of Consent.

## RESULTS

**Fig. 1:**
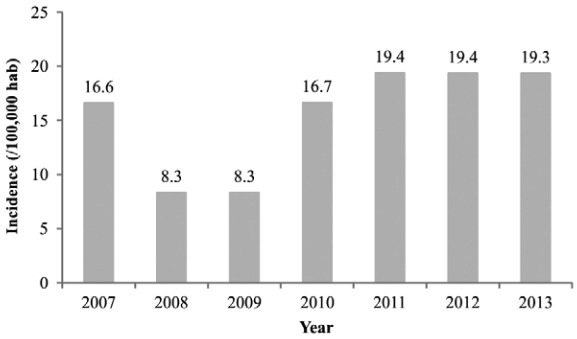
: incidence of visceral leishmaniasis between 2007-2013, in the city of Araçuaí, Minas Gerais (MG), Brazil. Source: population estimated from1991, 2000 and 2010 censuses data, and 1996 and 2007 population counts data from Instituto Brasileiro de Geografia e Estatística, and visceral leishmaniasis cases from the Municipal Secretary of Health of Araçuaí, MG, Brazil.

Between 2007-2013 a total of 41 cases of human VL were reported in Araçuaí, however, two (4.9%) of these were not analysed due to the fact that the addresses were incorrect, making it impossible to locate the individuals. During the study period, a decrease in the incidence of disease was observed in 2008 and 2009, followed by an increase in 2010, and stabilisation from 2011 on (Fig. 1).

The largest number of reported cases of VL was observed among children, mainly four years and younger, male individuals and individuals living in urban areas (Table I). However, the differences were significant for the sex variable (corrected *X* ^2^ = 6.564; p = 0.0104) and the sex between those older than 10 years, with a predominance of men in both cases (corrected *X* ^2^ = 4.05; p = 0.0442).

**TABLE I:** Profile of patients with visceral leishmaniasis in Araçuaí, Minas Gerais, Brazil, between 2007-2013. Source: individual notification forms of the municipal information system on notifiable diseases

Variables		Year							Total (%) (n = 39)

		2007 (n = 6)	2008 (n = 3)	2009 (n = 3)	2010 (n = 6)	2011 (n = 7)	2012 (n = 7)	2013 (n = 7)	
Sex	Male	4	2	2	4	4	7	5	28 (71,8%)
	Female	2	1	1	2	3	0	2	11 (28,2%)
Age (year)	0 - 04	3	1	2	1	0	1	4	12 (30,8%)
	04 - 10	2	0	1	1	0	1	2	7 (17,9%)
	10 - 20	0	1	0	2	1	3	0	7 (17,9%)
	20 - 39	0	1	0	1	1	2	0	5 (12,8%)
	39 - 60	1	0	0	1	2	0	0	4 (10,3%)
	> 60	0	0	0	0	3	0	1	4 (10,3%)
Zone of residence	Urban	5	2	1	5	2	4	6	25 (64,1%)
	Rural	1	1	2	1	5	3	1	14 (35,9%)

Regarding the activities they were performing at the time of discovery of infection among individuals over 18 years of age, eight worked in farming or planting, three were students, two were builder and three worked in general services.

With respect to case outcomes, 36 (92.3%) were cured and three (7.7%) resulted in death. One of these fatal cases had a coinfection with HIV.

In regards to canine infection, data from tests performed on 487 dogs were collected in 2013, 95 (19.5%) of which tested seropositive.

**Fig. 2:**
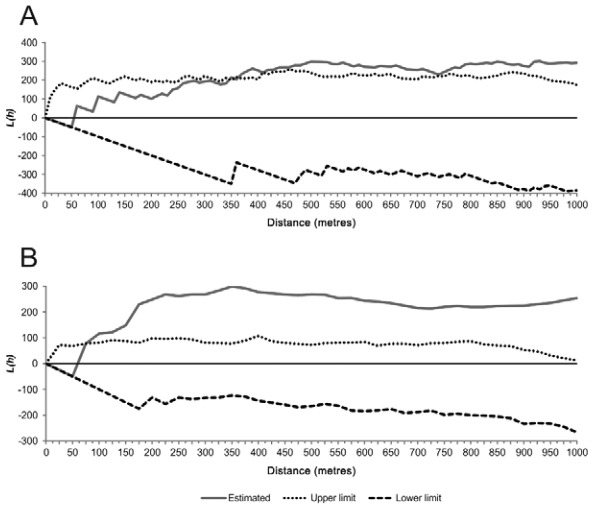
: graph of the K function expressed in L function values for the occurrence of visceral leishmaniasis in the city of Araçuaí, Minas Gerais, Brazil. (A) Human visceral leishmaniasis cases (2007-2013); (B) canine visceral leishmaniasis (2013).

The spatial distribution of human and canine VL in Araçuaí exhibited an aggregate pattern, with statistically significant groupings at distances greater than 350 m for human VL (Fig. 2A) and 75 m for canine infection (Fig. 2B).

**Fig. 3:**
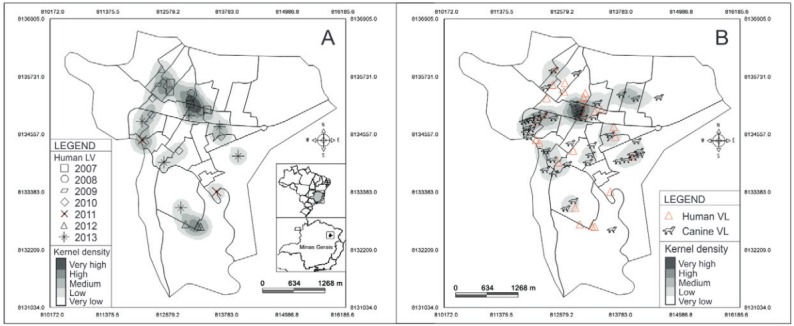
: kernel map of visceral leishmaniasis in the urban area of Araçuaí, Minas Gerais, Brazil. (A) Human visceral leishmaniasis (2007-2013); (B) canine visceral leishmaniasis (2013). Limits represent census lines according to the 2010 census.

The exploratory analysis using KDE points to a larger occurrence of cases in humans within the central area of the city (Fig. 3A). In a similar manner, canine infection was concentrated in those areas, with a clear overlap with areas of human infection (Fig. 3B). A strong positive correlation and statistical significance between densities of human and canine cases was observed (ρ = 0.6377; IC 95% = 0.6284 to 0.6469; p < 0.0001).

In relation to the density of occurrence of VL cases and NDVI, a negative correlation was observed for both human cases (ρ = -0.1921; IC 95% = -0.2071 to -0.1770; p < 0.0001) and canine cases (ρ = -0.2314; IC 95% = -0.2462 to -0.2166; p < 0.0001).

By analysing the socio-environmental conditions and residential structures of people who had VL in Araçuaí, it was found that the majority had a cement floor (66,7%) and brick walls with plaster (76,9%). Besides, the houses generally exhibited piped water, latrines for wastewater, and a covered water storage tank. Besides this, the majority of residences had a garden or backyard with shrubs and many fruit trees, the presence of various animals including dogs, cats and birds, proximity to natural environments and crowded houses close to one another. These and other socio-environmental characteristics of the housing conditions of individuals affected by VL are presented in Table II.

**TABLE II:** Socio-environmental characteristics and structures of household environments of people with visceral leishmaniasis between 2007-2013, in Araçuaí, Minas Gerais, Brazil

Variables	Categories	(n)	(%)
Piped water	Absent	7	18.0
	Present	32	82.0
Water and waste disposal	Open air	2	5.1
	Septic tank (Latrines)	26	66.7
	Sewer system	11	28.2
Home water storage	Covered water tank	23	59.0
	Uncovered water tank	6	15.4
	Cistern	4	10.2
	Containers	6	15.4
Number of residents in household	≥ 01 ≤03	9	23.1
	≥ 04 ≤ 06	23	59.0
	> 06	7	17.9
Distance to nearest neighbor	≤ 01metre	5	12.8
	> 01 ≤ 05 metres	23	59.0
	> 05 metres	11	28.2
Number of houses surrounding the investigated residence in a radius of 300 m	0	3	7.7
	≥ 01 ≤ 10	9	23.1
	≥ 11 ≤ 50	14	35.9
	> 50	13	33.3
Observation of gardens or backyard	Accumulated trash	4	10.5
	Shrubs	27	71.1
	Fruit trees	33	86.8
	Ornamental trees	2	5.3
	Garden absent	1	2.6
Frequency with which gardens and yards were cleaned	Daily	13	34.2
	Once per week	22	57.9
	Twice per month	3	7.9
Trash disposal	Collected once per week	5	12.8
	Collected twice per week	6	15.4
	Collected three times per week	16	41.0
	Disposed of outside	10	25.6
	Buried or burnt	1	5.2
Proximity to forest	No forest nearby	6	15.4
	100 to 200 metres	31	79.5
	200 to 500 metres	2	5.1
Presence of domestic animals	Dogs	32	82.0
	Cats	20	51.3
	Birds	33	84.6
	Pigs	18	46.2
	Horses	14	35.9
	Cattle	11	28.2
Education level of head of household	No schooling	5	12.8
	Incomplete primary education	29	74.3
	Complete primary education	3	7.7
	Complete high school	1	2.6
	Graduation	1	2.6

Among the 39 residences researched, eight (20.5%) were associated with previous occurrences of the disease in the family.

## DISCUSSION

In the city of Araçuaí, VL exhibits a pattern similar to that observed in the literature, with a higher prevalence in children primarily younger than four years and in male individuals (de Araújo et al. 2012, Góes et al. 2012). The high incidence of the disease in children is due to the fact that their immune systems are not yet fully developed (Missawa & Borba 2009). With respect to male individuals, it is believed that the higher incidence is related to hormonal factors present beginning in puberty (Guerra-Silveira & Abad-Franch 2013) and behavior, due to a greater exposure in adult men from labor activities (do Prado et al. 2011, Guerra-Silveira & Abad-Franch 2013, Ortiz & Anversa 2015).

The lethality of VL in Araçuaí (7.7%) during the period from 2007-2013 was higher than the national average (5.8%) between 2006-2009 (MS/SVS 2011). However, because it is a local study, a single death may have a proportionally greater impact on this rate. Moreover, since the evolution of the case is a data field not required at the national level, these data may be underreported. Of the people infected by VL who died, one had coinfection with HIV, a condition that has been increasingly observed (Barbosa et al. 2013, Carvalho et al. 2013, Ortiz & Anversa 2015, Távora et al. 2015) and is one of the causes of reduced survival of patients with this disease (de Sousa-Gomes et al. 2011, d, de Albuquerque et al. 2014).

The urbanisation of VL, found in Araçuaí by the predominance of cases in the central area of the city, as well as the observed negative correlation between NDVI and the density of human and canine cases, has been reported in other Brazilian cities (de Almeida et al. 2014, Vieira et al. 2014, Ortiz & Anversa 2015). This pattern is probably due to the success of *Lu. longipalpis* - already observed in Araçuaí in 2011 (SES-MG, unpublished observations) - in establishing itself in anthropic environments (Carvalho et al. 2010, Saraiva et al. 2011, Brazil 2013, Salomón et al. 2015), where there is a presence of domestic reservoirs and susceptible hosts.

The spatial overlap of cases of human and canine infection by *L. infantum* was already described in other locations (Margonari et al. 2006, Teixeira-Neto et al. 2014). However, besides this overlap, a strong correlation between the densities of human and canine infections was observed in this study, evidencing the importance of dogs in the occurrence of VL in this city. In this sense, the large number of dogs found in households of individuals who had VL in previous years highlights the need to maintain surveillance in these areas. The substitution of infected dogs that were eliminated for other susceptible ones allows these areas to continue to maintain the transmission cycle of *L. infantum* (Andrade et al. 2007).

The majority of sites of VL occurrence in the city showed deficiencies in sanitation infrastructure and crowded populations in poor living conditions with a large quantity of animals and organic material from shrubs, fruit trees, waste and garbage. Such conditions favor the maintenance of reservoirs in the areas surrounding homes and the proliferation of vectors (Bigeli et al. 2012, da Silva et al. 2012, Jeraldo et al. 2012, Barata et al. 2013, Belo et al. 2013, Costa et al. 2013, Coura-Vital et al. 2013, Vieira et al. 2014, Pimentel et al. 2015, Tanure et al. 2015).

Another relevant aspect to the epidemiology of VL is the level of education, which influences quality of life and health promotion by increasing the chance of greater access to information (Lima et al. 2010). In this study, VL mainly affected children living with heads of household who had completed only partial primary education. This, in addition to low income, may be associated with a higher incidence of disease among socially vulnerable populations (de Almeida et al. 2011, de Araújo et al. 2013, Ortiz & Anversa 2015). On the other hand, with the expansion of VL in urban areas, people and animals belonging to the middle and upper classes have also been infected, although to a lesser extent (de Almeida et al. 2010, Ortiz & Anversa 2015).

The fact that some people affected by VL have mentioned previous cases of the disease in the family probably indicates that they were exposed to the same risk factors. Therefore, it is necessary to invest in integrated programs for urban infrastructure, environmental management, vector and reservoir control, as well as promoting health education in areas where VL occurs, in order to ensure continued disease surveillance.

In recent years, VL has continued to expand into urban areas and a greater diversification of its epidemiological characteristics has been observed (Ortiz & Anversa 2015). In this context, local studies that integrate epidemiological data with spatial analysis tools help to understand the particularities of disease transmission dynamics, as well as assist in the definition of priority areas for the planning by competent agencies to reduce the incidence of infection.
